# Effects of Prebiotic Dietary Fibers on the Stimulation of the Mucin Secretion in Host Cells by In Vitro Gut Microbiome Consortia

**DOI:** 10.3390/foods13193194

**Published:** 2024-10-08

**Authors:** Seonghun Kim, Ji Young Kang, Quang Anh Nguyen, Jung-Sook Lee

**Affiliations:** 1 Jeonbuk Branch Institute, Korea Research Institute of Bioscience and Biotechnology, 181 Ipsin-gil, Jeongeup 56212, Republic of Korea; jiyoka@kribb.re.kr; 2 Department of Biosystems and Bioengineering, KRIBB School of Biotechnology, University of Science and Technology (UST), 217 Gajeong-ro, Daejeon 34113, Republic of Korea; quanganh@kribb.re.kr; 3 Korean Collection for Type Cultures, Korea Research Institute of Bioscience and Biotechnology, 181 Ipsin-gil, Jeongeup 56212, Republic of Korea; jslee@kribb.re.kr

**Keywords:** in vitro gut microbiome consortia, short-chain fatty acid, polysaccharide, dietary fibers, mucin, carbohydrate-active enzymes

## Abstract

The gastrointestinal microbiota are important for human health. Dietary intake may modulate the composition and metabolic function of the gut microbiome. We examined how the breakdown of prebiotic dietary fibers by the gut microbiome affects mucin secretion by intestinal epithelial cells. Metagenomic analyses of in vitro gut microbiome consortia revealed taxonomic profiles and genetic diversity of carbohydrate-active enzymes that digest polysaccharides. Two independent consortia exhibited different abilities to produce acetic acid, propionic acid, and butyric acid via the fermentation of polysaccharides derived from dietary fibers of grains and mushrooms. Although acetic acid generally had the highest concentration, the ratios of butyric acid and propionic acid to acetic acid varied depending on the polysaccharide source. These short-chain fatty acids affected morphological differentiation and mucin secretion in HT-29 human intestinal epithelial cells. These results suggest that prebiotic dietary fibers can be digested and metabolized by the gut microbiome to short-chain fatty acids, which can affect gut epithelial cells both directly and indirectly via the modulation of the gut microbiota and their enzymes.

## 1. Introduction

Polysaccharides, dietary fibers, and their derivatives can act as prebiotics that influence the composition of the gut microbiome and its metabolites [1,2]. Plant polysaccharides can be categorized into two groups, both of which serve as food and energy sources in humans. The first group is the storage carbohydrates found in most crops, such as starch and oligosaccharides. The second group is the non-digestible carbohydrates such as cellulose, hemicellulose, pectin, and glucan found in the cell walls of plants, algae, mushrooms, and yeast. Both groups can significantly affect human health [1,2,3].

Most storage carbohydrates can be digested by the endogenous hydrolyzing enzymes of the human gut, which convert them into monosaccharides such as glucose or galactose that rapidly cross the epithelial layer and are taken up in the organs. Other carbohydrates that are not digestible by human metabolic enzymes are metabolized by microbial consortia in the gastrointestinal tract [1,4]. These microbial consortia secrete diverse carbohydrate-active enzymes (CAZymes) to hydrolyze polysaccharides and oligosaccharides into metabolites that can be used by cells as carbon and energy sources [1,5]. Thus, the gut microbiome can break down non-digestible polysaccharides derived from dietary fibers into carbohydrate monomers or oligomers [1,2,6], which are subsequently metabolized further into short-chain fatty acids (SCFAs) such as butyric acid, propionic acid, and acetic acid [4,5,6]. By modulating the composition and metabolic function of the microbial communities in the gastrointestinal tract, non-digestible carbohydrates and their derivatives play important roles in human health and disease [1,5].

To date, many CAZymes have been identified, characterized, and analyzed from the genomes of individual bacteria isolated from the human gut [5,7]. Large-scale studies have been conducted to analyze microbiomes using meta-genomics, proteomics, and transcriptomics [8]; however, only a few CAZymes have been reported from such studies [9]. Heterogeneous carbohydrate hydrolases, combined with other sugar-modified enzymes that act on diverse polysaccharides, can be employed by microbiota to metabolize fermentable and non-fermentable carbohydrate compounds, causing changes in the intestinal levels of SCFAs, other organic acids, and amino acid derivatives [10], which can directly influence host cells as well as bacteria in the gut [5,11,12]. CAZymes secreted by the gut microbiome might also degrade mucin glycans and thus promote epithelial inflammation leading to inflammatory bowel disease [13]. However, the effects of microbiome-derived fermentation products on the glycoconjugates present on epithelial cells have not been characterized, and the overall diversity and numbers of CAZymes in the gut microbiome that can hydrolyze fermentable or non-fermentable polysaccharides remain unknown.

It is reasonable to hypothesize that the gut microbiome secretes various CAZymes to break down polysaccharides into oligo- and monosaccharides that are subsequentially metabolized to SCFAs, which affect the expression of mucins by intestinal epithelial cells. We tested this hypothesis by exploring the effects of metabolites produced by two independent in vitro gut microbiome consortia on the secretion of mucous glycoproteins in HT-29 human intestinal epithelial cells. In addition, we determined metagenomic CAZyme profiles for the consortia to evaluate the potential for SCFA production via polysaccharide fermentation. Our results confirm that polysaccharide metabolites produced by the gut microbiome can affect the quantity of mucin-type glycoproteins secreted from intestinal epithelial cells.

## 2. Materials and Methods

### 2.1. Materials

Levan, α-mannan, and pullulan were obtained from Sigma-Aldrich (St. Loius, MO, USA). β-(1,3(6))-glucan from yeast and β-(1,3(4))-glucans from barley and oat flour were purchased from Megazyme (Wicklow, Ireland). Mushrooms (*Hericium erinaceus* and *Sparassis crispa*), oats (*Avena sativa*), and hulless barley (*Hordeum vulgare*) were obtained from a local market in Korea.

### 2.2. Preparation of In Vitro Gut Microbiome Consortia

To study the gut microbiome of healthy Koreans, fresh stool samples were collected at the Bundang Seoul National Hospital (Korea). Isolation of gut microbiomes from the human fecal samples was approved by the Institutional Review Board of the Korea Research Institute of Bioscience and Biotechnology in Korea (P01-201702-31-007). The fecal samples were directly inoculated in brain heart infusion (BHI) broth. After a 24 h at 37 °C in an anaerobic chamber supplied with 90% N_2_, 5% CO_2_, and 5% H_2_, a sample of the pre-culture (10% *v*/*v*) was transferred to fresh culture broth and cultivated under equivalent conditions for 5 days. Cell growth and metabolite production were monitored. Once the in vitro microbiome consortia were stabilized, the final culture was aliquoted, prepared as frozen stock, and stored at −80 °C for further experiments.

### 2.3. DNA Extraction and Microbial Community Analysis

Total DNA was extracted from the in vitro microbiome consortia using an UltraClean Microbial DNA isolation kit (MoBio Laboratories Inc., Greater, CA, USA) according to the manufacturer’s instructions. The quality and quantity of extracted DNA were measured using Eppendorf BioSpectrometer fluorescence. The 16S rRNA genes were amplified by PCR using a primer set targeting the prokaryotic V4–V5 region. The amplified DNA was cleaned, quantified, and barcoded for construction of a DNA library using the Nextera^®^ XT index kit. The barcoded DNA libraries were sequenced using the Illumina Miseq platform (Illumina, San Diego, CA, USA) at MACROGEN (Seoul, Republic of Korea). The raw paired-end 16s rRNA sequences were used for taxonomical analysis according to our previous procedures [14].

### 2.4. Metagenome Shotgun Sequencing and Functional Annotation

Metagenomic DNA libraries of the in vitro gut microbiome consortia were constructed using the NEBNext^®^ UltraTM DNA Library Prep Kit (New England Biolabs, Ipswich, MA, USA). The paired-end libraries were sequenced using the PacBio RS II system as recommended by the manufacturer (PacBio, San Diego, CA, USA). de novo assembly of contigs was conducted using an iterative De Bruijn Graph de novo Assembler for Short Reads Sequencing Data with Highly Uneven Sequencing Depth. The sequence reads were mapped to the assembled contigs using the Burrows–Wheeler Transform and the Ferragina–Manzini index to confirm the number of reads used to assemble each contig. Genes were predicted using Prodigal in metagenomic mode, annotated using BLAST against the EggNOG database, and classified according to their function. In addition, Centrifuge was used for taxonomic classification and quantification of the assembled contigs. CAZyme prediction was conducted using the HMMER v3.3.2 package in dbCAN (https://bcb.unl.edu/dbCAN2/, accessed on 28 March 2018), running against Pfam Hidden Markov Models, and the eggnog database with the web-based eggnog-mapper (http://eggnog-mapper.embl.de/, accessed on 28 March 2018).

### 2.5. Production of SCFAs via Polysaccharide Fermentation

To evaluate SCFA production from polysaccharides by the in vitro gut microbiome consortia, we cultivated the consortia in fresh BHI broth for 24 h under anaerobic conditions and then transferred five independent samples of the cultures (1/100, *v*/*v*) to fresh media supplemented with various polysaccharides, each at a concentration of 1% (*w*/*v*). For the polysaccharide resources, ground powders of lyophilized mushrooms (*H. erinaceus* and *S. crispa*) and crops (oats and hulless barley) were used as food-derived polysaccharides, and α-mannan, levan, pullulan, β-(1,3(6))-glucan of yeast, and β-(1,3(4))-glucans of barley and oat flour were used as standard polysaccharides. Each culture was incubated at 37 °C for 120 h under stationary, anaerobic conditions. For SCFA quantification, the culture broth was centrifuged, and the cell-free supernatant was analyzed by high-performance liquid chromatography (HPLC).

### 2.6. SCFA Analysis

SCFAs were analyzed using an HPLC system equipped with a refractive index detector, an auto-sampler, and a Rezex ROA-Organic Acid H+ column (Phenomenex Inc., Torrance, CA, USA). All samples were clarified by filtration using a 0.20-μm filter and then injected into the analytical HPLC column. The column temperature was maintained at 65 °C. The mobile phase consisted of 0.005 N sulfuric acid with a flow rate of 0.5 mL min^−1^ under isocratic conditions. Under these conditions, hexanoic acid, propionic acid, formic acid, acetic acid, butyric acid, isobutyric acid, and valeric acid were detected at retention times of 3.92 min, 17.37 min, 18.96 min, 26.37 min, 34.99 min, and 35.12 min, respectively. All analyses were performed in triplicate.

### 2.7. HT-29 Cell Culture and Mucin Detection

HT-29 human colorectal adenocarcinoma cells were incubated at 37 °C with 5% (*v*/*v*) CO_2_ in a humidified incubator with Dulbecco’s modified essential medium (DEME) supplemented with 10% (*v*/*v*) fetal calf serum, penicillin (100 units/mL), and streptomycin (100 μg/mL) as the basal culture medium. When the cells reached 80–90% confluency, they were harvested, counted, and seeded at a density of 30,000 cells/mL (0.45 mL) per well in 24-well culture plates. The culture medium was replaced with basal medium containing approximately 5 mM SCFAs (total concentration of butyric acid, propionic acid, and acetic acid) in cell-free supernatants from culture broths of in vitro gut microbiome consortia supplemented with different polysaccharides. Prior to their use, the cell-free supernatants were titrated up to pH 7.0 and then filtered using a 0.2 μm filter. As a positive control, 1 μM methotrexate was used instead of SCFAs. On day 28, the cells were dissociated and harvested.

To detect secreted mucins, the adapted HT-29 cells were seeded in a 96-well back microplate (Greiner Bio-One GmbH, Kremsmünster, Austria) containing different concentrations of the cell-free supernatants. After the cells were grown for another 48 h, the culture medium was discarded. The cells attached to the plate were washed twice with PBS, fixed for 15 min in 4% paraformaldehyde prepared in PBS, and washed three times with 0.05% (*v*/*v*) Tween 20 in PBS (PBST). The cells were then blocked with 1% (*w*/*v*) bovine serum albumin in PBS for 1 h. To detect mucins, the plate was incubated at 4 °C overnight with 200 μg/mL Alexa Fluor 568-conjugated MUC2 and MUC5 antibodies (1:1000; Santa Cruz, Dallas, TX, USA). After the plate was washed three times with PBST, the quantities of mucins and core 1 O-glycans were measured in relative fluorescence units using the SynergyTM H1 Multi-Mode Reader (BioTek, Winooski, VT, USA). The fluorescent signals for MUC2/MUC5AC and rHEL2a were detected at Ex 544-Em 590 and Ex 485-Em 538, respectively, using an ELISA protocol [15]. In addition, cell proliferation was assayed using the Cell Counting Kit-8 (Dojindo Laboratories, Kumamoto, Japan), as per our previous procedures [16].

For confocal microscopy analysis, the adapted cells were seeded and cultivated for 48 h in 0.02% (*w*/*v*) poly-L-lysine-coated μ-dishes (Ibidi, Gräfelfing, Germany) containing DEME with 5 mM SCFAs from the cell-free supernatants. After the cells were washed and fixed as described above, they were incubated overnight with 200 μg/mL Alexa Fluor 568-conjugated MUC/MUC5 antibodies (1:1000) and then for 2 h with 200 μ/mL Alexa Fluor 488-conjugated rHEL2a lectin (1:500) [17]. The labeled cells were washed three times with PBST and analyzed using a Zeiss LSM 510 META Axiovert 200M ConfoCor 3 confocal microscope (Carl Zeiss MicroImaging GmbH, Jena, Germany).

### 2.8. Statistical Analysis

Data were analyzed by one-way or two-way analysis of variance using Sigma Plot 14 (Systat Software, Warrenton, VA, USA). *p*-values < 0.05 were considered statistically significant. Differences between groups were analyzed by Bonferroni’s multiple range test. The results are presented as the mean with standard errors for each variable.

## 3. Results and Discussion

### 3.1. Construction of the In Vitro Gut Microbial Consortia

Fecal samples from two individuals (samples 1 and 2) were separately inoculated in BHI broth and cultivated at 37 °C for 24 h under anaerobic conditions. Subsequently, 1% (*v*/*v*) of the pre-culture was transferred to fresh culture broth and cultivated for 5 days. To identify the microbial populations present in the in vitro consortia derived from samples 1 and 2 (consortia 1 and 2, respectively), the V4–V5 regions of 16S rRNA genes were PCR-amplified and sequenced. An analysis of eukaryotic communities was excluded because libraries based on ITS3–4 gene amplification could not be constructed owing to the extremely low contents of eukaryotic strains in both samples. In the bacterial community analysis, 269,714 and 280,082 paired-end reads for consortia 1 and 2 were generated, merged, and clustered into 178 and 173 operational taxonomic units (OTUs), respectively. Most (99.9%) of the OTUs in both consortia were attributed to a bacterial origin.

**Figure 1:**
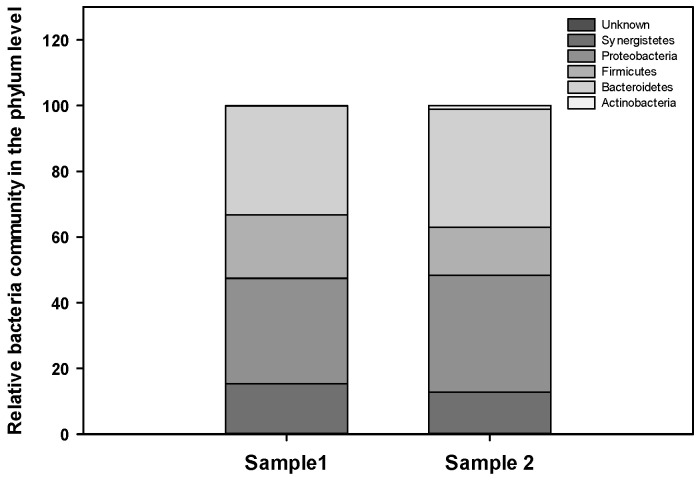
The taxonomic distribution of the in vitro microbiome consortia at the phylum level. The relative abundances of the phyla present in the consortia derived from samples 1 and 2 are shown as percentages.

In consortium 1, the taxonomy of OTUs comprised 5 phyla, 11 classes, 15 orders, 30 families, and 66 genera. The predominant phyla were Firmicutes (45.28%), followed by Proteobacteria (41.96%), Bacteroidetes (8.16%), Actinobacteria (4.54%), and a minor proportion classified as unknown (0.06%), which collectively accounted for 99.9% of all the 16S rRNA gene sequences (Figure 1). The taxonomy of OTUs in consortium 2 similarly included 5 phyla, 11 classes, 14 orders, 27 families, and 64 genera; however, the predominant phyla were Proteobacteria (49.21%), followed by Bacteroidetes (27.45%), Firmicutes (22.14%), Actinobacteria (1.13%), and a minor proportion classified as unknown (0.07%), which collectively accounted for 99.9% of all the 16S rRNA gene sequences (Figure 1).

The taxonomical compositions of the consortia showed a reduction in microbial diversity compared with that in the fecal samples 1 and 2 (Supplementary Data S1), presumably because of the selective pressure of abundant nutrients in the medium during in vitro cultivation. At the phylum level, the abundance of Bacteroidetes, such as the *Bacteroides* and *Phocaeicola* genera, was drastically enhanced in the in vitro consortia compared with that in the corresponding fecal samples. Moreover, the abundance of *Actinobacteria*, such as the *Bifidobacterium*, *Senegalimassilia*, and *Collinsella* genera, as well as Firmicutes, such as the *Enterococcus* and *Streptococcus* genera, was also increased in the vitro consortia compared with that in the corresponding fecal samples. At the genus level, the dominant bacterial genera in consortium 1 were *Klebsiella* (28.05%), *Escherichia* (12.22%), *Phocaeicola* (3.94%), *Bacteroides* (3.81%), *Peptoniphilus* (2.69%), *Clostridium* (2.42%), and *Enterobacter* (1.5%), whereas those in consortium 2 were *Citrobacter* (33.09%), *Bacteroides* (22.22%), *Sutterella* (7.95%), *Klebsiella* (4.01%), *Peptoniphilus* (3.75%), *Phocaeicola* (3.09%), *Pseudoflavonifractor* (2.87%), *Clostridium* (2.75%), *Enterobacter* (2.02%), and *Parabacteroides* (1.2%).

The abundance of Proteobacteria was marginally different between the two in vitro consortia. In a comparison between the two fecal samples, the abundances of the dominant genera *Acidovorax* and *Pseudescherichia* were relatively low in samples 1 and 2, respectively. Although the proportions of *Enterobacter*, *Escherichia*, and *Klebsiella* belonging to the Enterobacteriaceae family were higher in consortium 1 than in consortium 2, the members of the Enterobacteriaceae family were still predominant in both in vitro consortia, accounting for 41.8% in consortium 1 and 40.0% in consortium 2. These taxonomic analyses revealed the presence of diverse microorganisms within the in vitro consortia and showed that the diversity of the microbial communities in the fecal samples was modulated by the supplementation of specific nutrients in the culture medium [18].

### 3.2. Metagenome Analysis of CAZyme Genes in the In Vitro Consortia

The functional annotations assigned 25 subsystems in both metagenomes of the in vitro gut microbiome consortia. The annotations suggested that the consortia could produce a variety of secreted proteins with capacities to digest and metabolize dietary macromolecules. To investigate the potential of the consortia to enzymatically digest dietary fibers, we identified contigs encoding CAZymes and annotated them against the CAZy database using the dbCAN annotation algorithm. The resulting CAZyme profiles estimated the potential of the consortia to degrade polysaccharides derived from dietary fibers and the bioconversion of their derivatives. The identified CAZymes were classified as belonging to one of six families: glycoside hydrolases (GHs), glycosyltransferases (GTs), carbohydrate-binding modules (CBMs), auxiliary activities (AAs), carbohydrate esterases (CEs), and polysaccharide lyases (PLs) [5]. GHs, CBMs, and AAs are known as the main proteins for degrading polysaccharides [19].

**Figure 2:**
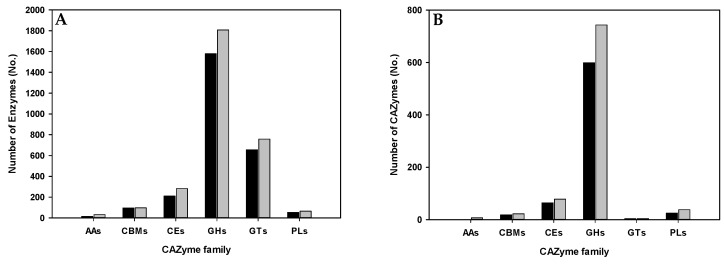
A comparison of predicted CAZymes between the two in vitro gut microbiome consortia. CAZymes were predicted based on the metagenome data from the consortia derived from samples 1 (black) and 2 (gray) using the CAZy database and the DIAMOND tool in dbCAN. (**A**) The numbers of total CAZymes functionally classified as auxiliary activities (AAs), carbohydrate-binding modules (CBMs), carbohydrate esterases (CEs), glycoside hydrolases (GHs), glycosyl transferases (GTs), and polysaccharide lyases (PLs). (**B**) The numbers of secreted CAZymes for each category in the in vitro gut microbiome consortia derived from samples 1 and 2.

Of the 97,747 and 46,104 coding sequences (CDSs) responsible for carbohydrate metabolism identified in the metagenomes of consortia 1 and 2, a total of 2611 (2.7%) and 3046 (6.6%) were identified as CAZymes with a cut-off E value of <1 × 10^−5^, respectively. The identified CAZymes included 1579 (60.5%) and 1807 (59.3%) GHs; 656 (25.1%) and 759 (24.9%) GTs; 211 (8.1%) and 282 (9.3%) CEs; 97 (3.7%) and 98 (3.2%) CBMs; 53 (2.0%) and 66 (2.2%) PLs; and 15 (0.6%) and 33 (1.1%) AAs in consortia 1 and 2, respectively (Figure 2A). Overall, CAZymes were more abundant in consortium 2 than in consortium 1. Relative to the number of GHs, the numbers of other CAZymes across both consortia were: GHs >> CEs > PLs > CBMs >> GTs > AAs. The relative abundances of CAZymes in the metagenomes suggest that hydrolysis of polysaccharides and their derivatives serves as the main enzymatic reaction for complex carbohydrate structures [20].

CAZymes with signal sequences that were predicted to be excreted by the bacteria displayed different patterns of abundance compared with the CAZymes overall (Figure 2B). Among the total predicted secreted proteins identified in consortia 1 and 2, 711 (27.2%) and 892 (29.3%) were CAZymes, respectively. Among these, 599 (84.2% of the total secreted proteins) and 743 (83.3% of the total secreted proteins) were GHs, 64 (9.0%) and 78 (8.7%) were CEs, 25 (3.5%) and 38 (3.9%) were PLs, 18 (2.5%) and 22 (2.5%) were CBMs, 4 (0.6%) and 4 (0.5%) were GTs, and 1 (0.1%) and 7 (0.8%) were AAs in consortia 1 and 2, respectively. The overall abundance profile for the secreted CAZymes was GHs >>> CEs >> PLs > CBMs >> GTs ≥ AAs. These results demonstrate that GHs were the predominant enzymes among the predicted extracellular proteins produced by the in vitro consortia for the digestion of various polysaccharides in dietary fibers. The consortia also had abundant genes for CEs and PLs, which assist GHs in polysaccharide degradation by modifying the polysaccharides [5,19].

### 3.3. Functional Prediction of GHs in the Metagenomes of the In Vitro Microbiome Consortia

**Figure 3:**
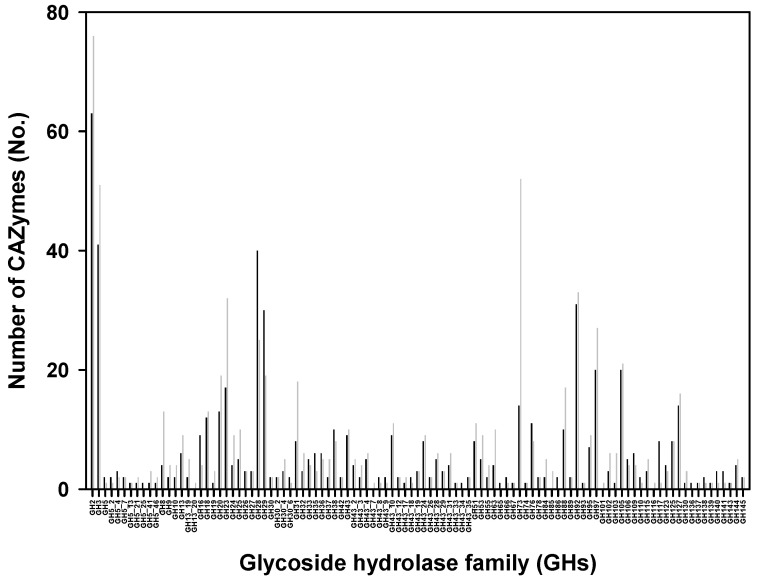
The numbers of secreted CAZymes belonging to different glycoside hydrolase GH families in the in vitro gut microbiome consortia derived from samples 1 (black bars) and 2 (gray bars).

Among the CAZymes previously identified the gut microbiome, GHs were the most dominant enzyme family for the hydrolysis of non-digestible polysaccharides in dietary fibers [10,20,21]. In our in vitro consortia, the highest numbers of secreted GHs were affiliated with GH family 2 (GH2), which accounted for 10.4% (63 genes) and 10.2% (76 genes) of the total secreted GHs in consortia 1 and 2, respectively (Figure 3; Supplementary Data S2; Table S1). Six other GH families (GH3, GH28, GH29, GH92, GH97, and GH105) were also highly abundant in both consortia. In addition, 20 families of GHs (GH2, GH3, GH13, GH18, GH20, GH27, GH29, GH30, GH31, GH32, GH35, GH36, GH38, GH42, GH43, GH92, GH95, GH97, GH116, and GH130) for mainly the hydrolysis of oligosaccharides were dominant and together accounted for 54.6% and 51.0% of the total GHs in consortia 1 and 2, respectively. In terms of the degradation of polysaccharides within dietary fibers, the hydrolases for starch degradation (GH3, GH13, GH13_19, and GH13_20) were 7.7% and 8.9% of the total hydrolases in consortia 1 and 2, respectively.

These families include α-amylase and disbranched hydrolases with hydrolytic activities on starch, amylose, and amylopectin that contain α-glucoside linkages [22,23]. The hydrolases for the hydrolysis of β-glucans, which contain β-glucoside linkages, were identified as GH16 for β-1,3/4-glucanase, GH30 for β-1,6-glucanase, and GH55 for exo/endo-β-1,3-glucanase; however, the abundances of these enzymes were lower than that of α-glucanase (2.1% and 1.3% in consortia 1 and 2, respectively).

For the degradation of hemicellulose consisting of xylan, arabinan, and galactan, GH43 and its 19 sub-families accounted for 11.0% and 10.2% of the total hydrolases in consortia 1 and 2, respectively. Although their abundances were much lower than those of the GH3 family, several other pectin degradation enzymes for rhamnogalacturonan I (GH28, GH35, GH53, GH78, GH93, and GH105) and rhamnogalacturonan II (GH137, GH138, GH139, GH140, GH141, and GH143) were detected in both consortia [24]. All the GH families involved in rhamnogalacturonan II degradation were identified as *Bacteroides* or *Bifidobacterium* enzymes [24]. GH28 and GH29 were the most abundant enzymes for pectin hydrolysis among the GH families. For the hydrolysis of xylan and xyloglucan and its derivatives in hemicellulose, GH10, GH31, and GH115 were detected at relatively low abundances among the secreted enzymes. The GH16, GH51, and GH74 families may be among the enzymes that can hydrolyze both xyloglucan and cellulose. These GH families function to help bacteria deconstruct the various polysaccharides of plant dietary fibers and generate fermentable sugars [21].

For the degradation of minority polysaccharides, enzymes to hydrolyze complex structures with various glycosidic linkage types were present to degrade mannan or galactomannan. Anomericity was also observed among the secreted CAZymes: GH26 exo-β-mannanases and GH130 endo-β-mannanases were identified with respect to β-mannan [25]. GH76 α(1,6)-mannanase, GH92 α1,(2,3,4,6)-mannosidase, and GH125 exo-α(1,6)-mannosidase were also confirmed with respect to α-mannan. Among these, the GHs for β-mannan hydrolysis accounted for less than 0.8% of the secreted GHs in both samples. Nevertheless, the hydrolytic enzymes acting on α-mannan accounted for 8.2% and 6.6% of the secreted GHs in consortia 1 and 2, respectively. In both consortia, the exo-acting GH92 family containing various α-mannosidases was extremely abundant (4.4–5.1% of the total secreted GHs), compared with other mannoidases. Among the mannanses, endo-acting GH76 and exo-acting GH92 can synergistically break down both α-mannan from *Saccharomyces cerevisiae* and high-mannose N-glycans from host cells [26].

In addition to the GH families, noncatalytic CBMs are present in microbes to increase binding specificities and efficiencies for carbohydrate degradation [27]. Among the total secreted proteins, 76 and 169 CBMs (CBM alone or CBM with other CAZymes) were observed in consortia 1 and 2, respectively (Table 1).

Consortium 1 showed less abundance and diversity for the CBM families than consortium 2 (consortium 1 contained 12 CBM families; consortium 2 contained 22 CBM families). The most dominant CBMs were CBM50 >> CBM32 > CBM67 in both consortia. These proteins can harbor binding affinities for chitin or peptidoglycan (CBM50), galactose and lactose (CBM32), and L-rhamnose (CBM67). Other CBMs can be classified into four groups based on their binding substrates: chitin-binding proteins (CBM5, CBM12, CBM55, and CBM73); xylan- and glucan-binding proteins (CBM4, CBM6, CBM9, CBM22, and CBM54); mannan-, galactomannan-, arabinogalactan-, and inulin-binding proteins (CBM23, CBM35, CBM38, CBM61, and CBM62); and starch- and α-glucan-binding proteins (CBM34 and CBM41/48). Additionally, CBM40 and CBM51, which bind sialic acid and galactose, respectively, of the blood group A/B antigens, would also bind to the glycans present on mucin and were detected in consortium 2 [28,29]. These results suggest that in vitro microbial consortium 2 might hydrolyze or convert polysaccharides and their derivatives into fermentable sugars partially via the action of various CBM proteins, in contrast to consortium 1.

### 3.4. Comparison of the Fermentation Capabilities for SCFA Production

**Figure 4:**
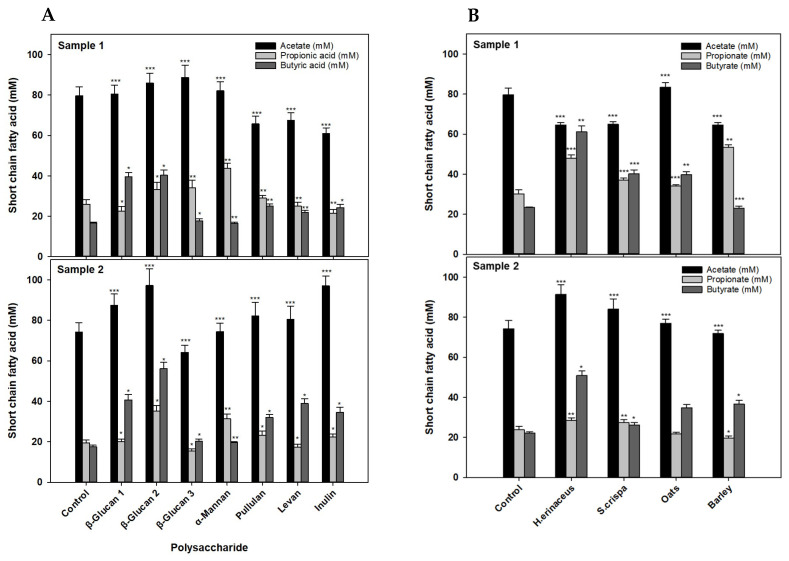
The production of different short-chain fatty acids by the in vitro gut microbiome consortia in BHI broth supplemented with 1% (*w*/*v*) β-glucans, α-mannan, pullulan, levan, or inulin (A) and with 1% (*w*/*v*) dried dietary fibers derived from mushrooms (*H. erinaceus* or *S. crispa*) and crops (oats or barley) (B). (mean ± SD, *n* = 5). *: *p* < 0.05; **: *p* < 0.01; ***: *p* < 0.001.

To test the abilities of the in vitro gut microbiome consortia to ferment different polysaccharides, we measured the levels of acetic acid, butyric acid, and propionic acid in culture media after the consortia were incubated for 120 h in culture broth supplemented with 1% (*w*/*v*) various polysaccharides. In comparison with control cultures without polysaccharide supplementation, both consortia increased the amounts of butyric acid and other SCFAs in cultures containing β-glucan 1 (barley) and β-glucan 2 (oats) (Figure 4A).

To further analyze SCFA production from food-derived polysaccharides by the consortia, we incubated the consortia with dried mushrooms (*H. erinaceus* or *S. crispa*) or crops (ground oats or hulless barley) instead of defined polysaccharides under the same culture conditions. These food substances are well-known materials that are rich in polysaccharides—such as β-glucan—rather than starch [30,31]. Mushroom species in particular are rich bioresources of potential prebiotics because of their polysaccharides and other bioactive compounds [32]. In control cultures without mushroom or crop supplementation, acetic acid was the most abundantly produced SCFA (Figure 4B).

The ratio of propionic acid to butyric acid varied among the tested substances. Nevertheless, compared with the controls, the production of propionic acid and butyric acid was mostly increased in the cultures incubated with the supplements, despite variability in the concentration of acetic acid. The production of butyric acid was particularly enhanced in most of the supplemented cultures, especially those incubated with *H. erinaceus*. In the preliminary measurements, the total glucan contents in dried *H. erinaceus* and *S. crispa* were 15.6 ± 1.3% (*g*/*g*) and 34.8 ± 0.5% (*g*/*g*), respectively.

Although *H. erinaceus* contained less glucan than *S. crispa*, both consortia showed high butyric acid production and less propionic acid production in the fermentation of these mushrooms. These results indicate that the amounts of SCFAs produced may not depend on the glucan content alone. Variation in SCFA production might be influenced by the fermentative abilities of different microbial consortia for different types of substances [3,33]. Moreover, the complexities of polysaccharides, which consist of homo- or hetero-type sugars with different glycosidic linkages, might also be a critical factor in the generation of fermentable sugars by diverse GHs [5,10,33]. In any case, our data clearly suggest that extra polysaccharides in the culture medium increased the amounts of SCFAs produced via microbiome metabolism.

### 3.5. The Effect of SCFAs on Mucin Secretion and Morphological Changes in HT-29 Cells

**Figure 5:**
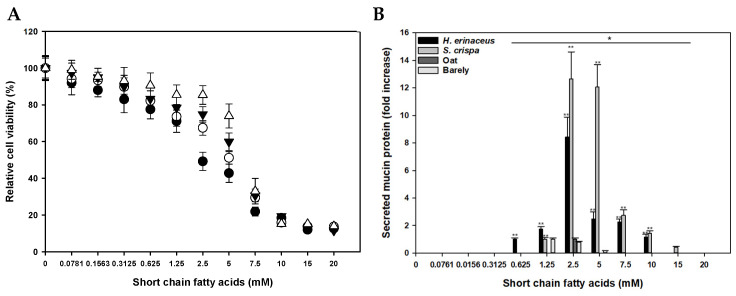
Short-chain fatty acids produced by the in vitro gut microbiome consortia suppressed proliferation and promoted mucin secretion in HT-29 cells. (A) The proliferative activity of HT-29 cells reported as mitochondrial redox potential in the presence of different concentrations of total short-chain fatty acids contained in bacteria-free supernatant from cultures of in vitro gut microbiome consortia (sample 1) supplemented with *H. erinaceus* (closed circles), *S. crispa* (open circles), oats (closed reverse triangles), or barley (open triangles). (B) Increases in mucin secretion by HT-29 cells after 2 days of incubation with different concentrations of total short-chain fatty acids in bacteria-free supernatant from cultures of in vitro gut microbiome consortia supplemented with *H. erinaceus*, *S. crispa*, oats, or barley. The secreted mucin proteins were detected using the fluorescent anti-human MUC2/MUC5AC antibodies. The fold increases are based on the lowest signal of each sample (mean ± SD, *n* = 3). *: *p* < 0.05; **: *p* < 0.01.

SCFAs have been reported to regulate cell proliferation, differentiation, apoptosis, and gene expression in intestinal in vitro cell models [33,34]. We analyzed the effects of bacterial SCFAs on proliferation, mucin secretion, and morphological changes in HT-29 human intestinal epithelial cells. We stimulated HT-29 cells with increasing concentrations (0.156 mM to 20 mM) of total SCFAs, which included acetic acid, propionic acid, and butyric acid, in cell-free supernatants of culture broths of the in vitro consortia (sample 1) supplemented with *H. erinaceus* (sample A), *S. crispa* (sample B), oats (sample C), or barley (sample D). After 48 h, the SCFA treatments inhibited HT-29 cell proliferation in a dose-dependent manner, which was confirmed by a cell proliferation assay (Figure 5A).

Furthermore, the results suggested that the specific organic acid composition of the SCFAs influenced the effect of the SCFAs on HT-29 cell viability. When the SCFAs were applied to HT-29 cells at a concentration of 5 mM, the SCFAs derived from bacterial cultures supplemented with *H. erinaceus* inhibited HT-29 cell growth more than those from bacterial cultures supplemented with other sources of polysaccharides. The SCFAs derived from cultures supplemented with *H. erinaceus* contained the highest concentration of butyric acid among the tested SCFA treatments. Additionally, the inhibition of HT-29 cell growth was correlated with the amount of butyric acid in the SCFA treatment. Although acetic acid was the most abundant organic acid in all the SCFA treatments, our results suggest that butyric acid is the most effective organic acid for suppressing HT-29 cell proliferation.

To measure the levels of secreted mucin, we detected MUC2/MUC5AC glycoproteins by ELISA in the culture supernatants of HT-29 cells adapted with different concentrations of SCFAs. Mucins were detectable in the supernatants of HT-29 cells treated with SCFA concentrations ranging from 0.625 mM to 15 mM (Figure 5B). No mucins were detected in supernatants of non-adapted HT-29 cells or HT-29 cells treated with SCFA concentrations less than 0.625 mM or more than 15 mM. The optimum concentration for mucin secretion was 2.5 mM SCFAs in most samples, except for the samples treated with SCFAs from cultures supplemented with barley. The SCFAs derived from cultures supplemented with *H. erinaceus* or *S. crispa* were more effective at inducing mucin secretion than those derived from cultures supplemented with oats or barley. The treatment of HT-29 cells with 2.5 mM or 5 mM SCFAs derived from cultures supplemented with *S. crispa* increased mucin secretion more than 12-fold. When the HT-29 cells were treated with the SCFAs derived from cultures supplemented with *H. erinaceus*, mucin production was increased 8-fold at an SCFA concentration of 2.5 mM compared with that in the untreated control. However, the mucin secretion induced by 2.5 mM SCFAs derived from cultures supplemented with *H. erinaceus* or *S. crispa* declined by 75% at SCFA concentrations of 5 mM and 7.5 mM, respectively. These results show that the SCFA mixtures enhanced mucin secretion by HT-29 cells at low concentrations, but had the opposite effect at high concentrations because of cell proliferation or apoptosis [35]. Indeed, mucin secretion could be regulated by the variable compositions of butyric acid, acetic acid, and propionic acid in the SCFA mixtures [35].

**Figure 6:**
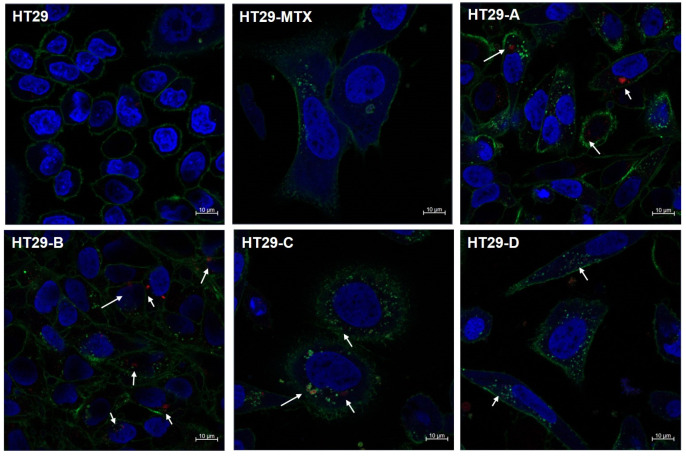
The detection of core 1-derived mucin-type O-glycans and secreted mucins in differentiated HT-29 cells using fluorescent rHEL2a lectin (green), anti-human MUC2/MUC5AC antibodies (red), and DAPI (blue). HT29: untreated negative control; HT29-MTX: methotrexate-stimulated cells (positive control); HT29-A, -B, -C, and -D: HT-29 cells exposed to short-chain fatty acids in bacteria-free supernatant from cultures of in vitro gut microbiome consortia supplemented with *H. erinaceus* (HT29-A), *S. crispa* (HT29-B), oats (HT29-C), or barley (HT29-D). The arrows indicate accumulated mucins detected by the mucin antibodies. The images were obtained using a confocal laser microscope.

To observe morphological changes in HT-29 cells, we incubated the cells with the cell-free SCFAs and then with Alexa Fluor 488-conjugated rHEL2a lectin for core 1-derived mucin-type O-glycan and with Alexa Fluor 568-MUC2/MUC5AC antibodies for mucin. The fluorescence-labeled cells showed morphological differentiation after cultivation with the SCFAs (Figure 6). Each SCFA treatment induced enlargement, elongation, and stretching of HT-29 cells, which took on an irregular shape similar to that of methotrexate-stimulated cells in comparison with untreated, roughly round negative control cells. In addition, phase-contrast microscopy showed that most of the HT-29 cells incubated with SCFAs were spindle-shaped and arranged in whorls, rather than round as in normal cells (Supplementary Data S3, Figure S1). Under confocal microscopy, the cells incubated with SCFAs displayed a larger cell size within a polarized nucleus, stained by DAPI, compared with untreated cells. Bacterial SCFAs can drive HT-29 cells toward goblet cell differentiation [36]. In the fluorescence-labeled cells, green fluorescence signals for the core 1 mucin-type O-glycans were predominantly observed on the cell surface, whereas red fluorescence signals for MUC2/MUC5AC proteins were much weaker and located in the cytosol. Nonetheless, the HT-29 cells incubated with SCFAs displayed stronger green fluorescence on their surface than untreated HT-29 cells. The difference in fluorescence intensities might be caused by the fact that the rHEL2a lectin can detect the core 1 mucin-type O-glycans present in all mucin types and other glycoproteins, whereas the mucin antibodies just detect two types of mucins for MUC2/MUC5AC proteins. Moreover, some green fluorescence was also detected as trafficking lectins moving near the nucleus in the cytosol of the cells, while minor red fluorescence for the mucin proteins was detectable in the cytoplasm. Additionally, the two fluorescence signals did not completely overlap each other, suggesting that the lectin and the antibodies could recognize different epitopes in mucins. Therefore, further studies are needed to evaluate mucin secretion/accumulation, morphological changes, and their dominant glycan structures in HT-29 after exposure to bacterial SCFAs.

## 4. Conclusions

We characterized two independent gut microbiome consortia with different abilities to ferment various polysaccharides derived from dietary fibers. The consortia had different taxonomic profiles and genetic diversity of CAZymes that digest polysaccharides. The consortia displayed unique capabilities to produce SCFAs derived from various polysaccharides and food resources. Although acetic acid was the main organic acid, the ratios of propionic acid and butyric acid to acetic acid in cultures containing the consortia were variable depending on the feed substances supplied to the cultures. The high content of butyric acid among the SCFAs produced by the gut microbiome consortia was especially effective at inducing morphological differentiation and mucin secretion in HT-29 intestinal epithelial cells. However, the limitation of this study is the in vitro cell experiments for mucin secretion by bacterial SCFAs using selected gut microbiome consortia. Further studies are needed to evaluate these events by in vivo experiment using an animal model with long-term polysaccharide feeding. These results highlight that independent gut microbiomes have different abilities to metabolize diverse polysaccharides derived from food resources, ultimately enhancing SCFA levels and providing health benefits by positively affecting intestinal cells in gut niches.

## Figures and Tables

**Table 1:** Prediction of the carbohydrate binding modules (CBMs) in the secreted CAZymes of the gut microbiome consortia in the CAZy database using the HMMER tool in dbCAN.

No.	Carbohydrate Binding Module	CBM (Number)		Potential Target Substrates
	(CBM)	Sample 1	Sample 2	
1	CBM5	2	-	Chitin
2	CBM6	2	2	Cellulose, β(1,4)-Xylan, β(1,3)-Glucan, β(1,3(4))-Glucan, and β(1,4)-Glucan
3	CBM9	3	-	Cellulose, Xylan
4	CBM13	1	1	GalNAc
5	CBM20	6	3	Starch, Cyclodextran
6	CBM26	-	1	Starch
7	CBM32	15	7	Galactose, Lactose, Lactosamine, Polygalacturonic acid
8	CBM34	5	17	Starch (granular)
9	CBM35	1	1	Xylan, Mannan, Mannooligosaccharides. Galactan
10	CBM38	2	-	Inulin (cycloinulo-oligosaccharide)
11	CBM40	1	1	Sialic acid
12	CBM41	1	3	α-Glucans amylose, Amylopectin, Pullulan
13	CBM48	10	24	Glycogen
14	CBM50	3	5	Chitin, Peptidoglycan, Chitopenta
15	CBM51	4	5	Galactose, Blood group A/B-antigens
16	CBM54	1	2	Xylan, Yeast cell wall glucan, Chitin
17	CBM57	1	-	Glc2-N-glycan
18	CBM58	1	-	Maltoheptaose
19	CBM62	2	4	Xyloglucan, Arabinogalactan, Galactomanna (Gal-minding)
20	CBM67	26	22	L-Rhamnose
21	CBM73	1	3	Chitin

## Data Availability

The data presented in this study are available on request from the corresponding author (The data are not publicly available due to privacy).

## Supplementary Materials

The following supporting information can be downloaded at: https://www.mdpi.com/article/10.3390/foods13193194/s1, Supplementary Data S1: Taxonomy abundance ratios in samples 1 and 2; Supplementary Data S2: Table S1. The prediction of glycoside hydrolases among the secreted CAZymes of the gut microbiome consortia; Supplementary Data S3: Figure S1. Phase-contrast microscopy analysis of SCFA-adapted HT29 cells.
