# Supplementary material for: Effects of Prebiotic Dietary Fibers on the Stimulation of the Mucin Secretion in Host Cells by In Vitro Gut Microbiome Consortia
Source: Foods. 2024 Oct 8;13(19):3194. doi: 10.3390/foods13193194 (PMC11475894; doi:10.3390/foods13193194)
Supplement: Supplementary file 1 [file foods-13-03194-s001.zip › Supplementary data 2.docx]

**Supplementary data 2**

**Table S1.** Prediction of the glycoside hydrolases (GHs) in the secreted CAZymes of the gut microbiome consortia

| No. | Glycoside hydrolyase (GH) | GH (Number) | | Potential target substrates |
| --- | --- | --- | --- | --- |
|  |  | Sample 1 | Sample 2 |  |
| 1 | GH2 | 63 | 76 | β-Gal; β-Man; β-D-glucuronic acid; α-L-arabinofuranoside; endo/exo-β-Man; β-GlcNAc; β-galacturonic acid; β-xyl; β-D-galactofuranoside; β-Glc; glycyrrhizin |
| 2 | GH3 | 41 | 51 | β-Glucan; Xylan; Glucosylceramidase; Hemicellulose; Cellulose; N-glyan; oligoxyloglucan; coniferin; xyloglucan; stevioside; protodioscin; Pectin; avenacin; tomatine |
| 3 | GH5 | 2 | 1 | Cellulose; Hemicellulose; Xylan; β-Mannan; β-glucosylceramide; cellodextrin; β-Glucan; cellobioside; Glycoceramide; β-primeveroside; xyloglucan; Galactan; Arabinoxylan; laminarin; chitosan; β-D-galactofuranoside; β-galactosylceramide; β-rutinoside; Rhamnose; Glucomannan; hesperidin |
| 4 | GH5_2 | 2 | 1 | Chitosan; β-glucan; β-xylan |
| 5 | GH5_4 | 3 | 2 | Glucomannan; oligoxyloglucan; xyloglucan; β-mannan |
| 6 | GH5_7 | 2 | 2 | β-mannan |
| 7 | GH5_13_ | 1 | 1 | β-D-galactofuran;α-L-arabinofuran |
| 8 | GH5_21 | 1 | 2 | β-xylan |
| 9 | GH5_25_ | 1 | - | β-Glucan; β-Mannan; β-Xylan |
| 10 | GH5_41 | 1 | 3 | β-mannan |
| 11 | GH5_46 | 1 | 2 | β-Glucan |
| 12 | GH8 | 4 | 13 | Chitosan; cellulose; lichenin; β-xylan; β-glucan; laminarin |
| 13 | GH9 | 2 | 4 | β-Glucan; Lichenin; Laminarin; cellodextrin; xyloglucan; β-glucosamine; xanthan |
| 14 | GH10 | 2 | 4 | arabinoxylan |
| 15 | GH13 | 6 | 9 | Starch; Glycogen |
| 16 | GH13_19 | 2 | 5 | maltooligosaccharide |
| 17 | GH13_26 | - | 1 | β-Glucan |
| 18 | GH16 | 9 | 4 | keratan-sulfate; laminarin; β-glucan; β-agarose; κ-carrageen; xyloglucan; galactan |
| 19 | GH18 | 12 | 13 | Chitin; peptidoglycan; chitobioside |
| 20 | GH19 | 1 | 3 | Chitin |
| 21 | GH20 | 13 | 19 | lacto-N-biosidase; mucin; sulfomucin |
| 22 | GH23 | 17 | 32 | Peptidoglycan; chitin |
| 23 | GH24 | 4 | 9 | Peptidoglycan |
| 24 | GH25 | 5 | 10 | Peptidoglycan |
| 25 | GH26 | 3 | 3 | β-mannan; β-xylan; lichen; β-glucan; mannobiose |
| 26 | GH27 | 3 | 3 | Galactan; isomalto-dextran |
| 27 | GH28 | 40 | 25 | Polygalacturon; rhamnogalacturon; galacturon; xylogalacturon |
| 28 | GH29 | 30 | 19 | Fucosylated N-glycan, O-glycan |
| 29 | GH30 | 2 | 2 | β-xylan; β-glucan; Pectin; glucosylceramide; galactan |
| 30 | GH30_2 | 2 | 2 | β-xylan |
| 31 | GH30_4 | 3 | 5 | β-D-fucoside |
| 32 | GH30_6 | 2 | 1 | β-glucoside |
| 33 | GH31 | 8 | 18 | Sucrose; maltose; maltooligosaccharide; isomaltose; α-xylose; α-xylan; sulfoquinovosyl diacylglyceride |
| 34 | GH32 | 3 | 6 | Iinulin; Fructan; levan; sucrose |
| 35 | GH33 | 5 | 4 | Sialoglycoconjugate |
| 36 | GH35 | 6 | 3 | Lactose; β-galactan; arabinogalactan; chitosan |
| 37 | GH36 | 6 | 5 | Stachyose; raffinose |
| 38 | GH37 | 2 | 5 | Trehalose |
| 39 | GH38 | 10 | 8 | Mannylated glycoproteins |
| 40 | GH42 | 2 | 2 | Lactose; galactooligosaccharides; galactans |
| 41 | GH43 | 9 | 10 | Arabinan; xylan |
| 42 | GH43_2 | 4 | 5 | α-L-arabinofuranoside |
| 43 | GH43_3 | 2 | 4 | β-D-galactofuranoside |
| 44 | GH43_4 | 5 | 6 | α-1,5-L-arabinofuranoside; arabinan |
| 45 | GH43_7 | - | 1 | xylan |
| 46 | GH43_8 | 2 | 1 | β-D-galactofuranoside |
| 47 | GH43_9 | 2 | 1 | α-L-arabinofuranoside |
| 48 | GH43_10 | 9 | 11 | Xylan; α-L-arabinofuranoside |
| 49 | GH43_12 | 2 | 2 | Xylan; α-L-arabinofuranoside |
| 50 | GH43_17 | 1 | 2 | Xylan; α-L-arabinofuranoside |
| 51 | GH43_18 | 2 | 1 | α-L-arabinofuranoside |
| 52 | GH43_19 | 3 | 3 | α-L-arabinofuranoside |
| 53 | GH43_24 | 8 | 9 | β-1,3-galactanase |
| 54 | GH43_26 | 2 | 2 | α-1,5-L-arabinofuranoside; α-L-arabinofuranoside |

*(Continue)*

| No. | Glycoside hydrolyase (GH) | GH (Number) | | Potential target substrates |
| --- | --- | --- | --- | --- |
|  |  | Sample 1 | Sample 2 |  |
| 55 | GH43_28 | 5 | 6 | α-1,2(3)-L-arabinofuranoside; β-1,4-xyloside |
| 56 | GH43_29 | 3 | 3 | α-1,2(4)-L-arabinofuranoside; α-L-arabinofuranosidase; xylan; |
| 57 | GH43_31 | 4 | 6 | β-D-galactofuranoside |
| 58 | GH43_33 | 1 | 1 | α-L-arabinofuranosidase |
| 59 | GH43_34 | 1 | - | β-D-galactofuranosidase;α-L-arabinofuranosidase |
| 60 | GH43_35 | 2 | 2 | xylan; α-L-arabinofuranoside |
| 61 | GH51 | 8 | 11 | L-arabinofuranoside; hemicelluloses (arabinoxylan, arabinogalactan, L-arabinan, etc.) |
| 62 | GH53 | 5 | 9 | β-1,4-galactan |
| 63 | GH55 | 2 | 4 | β-1,3-glucans, β-1,3(6)-glucans; laminarin; gentiobiose |
| 64 | GH63 | 4 | 10 | α-glucosidase in N-glycan; Nigerose (Glc-α-1,3-Glc); mannosylglycerate; glucosylglycerate |
| 65 | GH65 | 1 | - | maltose (Glc-α-1,4-Glc); trehalose (Glc-α1,α1-Glc); kojibiose (Glc-α-1,2-Glc); trehalose 6-phosphate (Glc-α1,α1-Glc6P) |
| 66 | GH66 | 2 | 1 | Isomaltooligosaccharides; dextran |
| 67 | GH67 | 1 | 1 | Glucuronic acid in xylooligosaccharides; xylan |
| 68 | GH73 | 14 | 52 | N-acetylglucosaminyl (NAG) and N-acetylmuramyl (NAM) moieties in bacterial peptidoglycans |
| 69 | GH74 | 1 | 1 | Xyloglucans; xyloglucan-oligosaccharides |
| 70 | GH76 | 11 | 8 | α-1,6-mannans (fungal mannoproteins, mycobacterial cell wall lipomannan, lipoarabinomannan and phosphatidylinositol mannosides) |
| 71 | GH78 | 2 | 2 | L-rhamnosides, including: flavonoid glycosides such as naringin, hesperidin and rutin; polysaccharides such as rhamnogalacturonan and arabinogalactan-protein and glycolipids |
| 72 | GH84 | 2 | 5 | hyaluronan |
| 73 | GH85 | - | 3 | chitobiose core (GlcNAc-β-1,4-GlcNac) of N-linked glycans |
| 74 | GH86 | 2 | - | β-1,4 glycosidic bonds of agarose; β-(1,3)-D-galactose (G) and 1,4-linked 3,6-anhydro-α-L-galactose (LA) residues of agarose |
| 75 | GH88 | 10 | 17 | unsaturated glucuronyl glycosides |
| 76 | GH89 | 2 | 2 | α-D-GlcNAc-(1,4)-D-Galactose in mucin; heparan sulfate; mucopolysaccharidosis |
| 77 | GH92 | 31 | 33 | yeast cell wall type mannose-1-phosphate-6-mannosides; α-mannan |
| 78 | GH93 | 1 | 1 | α-1,5-L-arabinan |
| 79 | GH95 | 7 | 9 | α-Fuc-1,2-Gal linkages of oligosaccharides; arabinoxylans; human milk oligosaccharides (2'-fucosyllactose); blood group glycoconjugates (ABO and Lewis antigens); xyloglucan (α-L-Gal-1,2-Xyl linkages) in corn glucuronoarabinoxylan |
| 80 | GH97 | 20 | 27 | α-linked D-glycosides in maltooligosaccharide (from maltose to maltoheptaose) |
| 81 | GH101 | - | 1 | disaccharide Gal-beta-1,3-GalNAc-alpha-R in Core 1 O-glycans on proteins (mucin) |
| 82 | GH102 | 3 | 6 | β-1,4-linkage between N-acetylmuramoyl and N-acetylglucosaminyl residues in peptidoglycan |
| 83 | GH103 | 7 | 6 | β-1,4-linkage between N-acetylmuramoyl and N-acetylglucosaminyl residues in peptidoglycan |
| 84 | GH105 | 20 | 21 | unsaturated glucuronyl/galacturonyl polysaccharides (rhamnogalacturonan-I; ulvan; arabinogalactan) |
| 85 | GH106 | 5 | 4 | L-Rha-α-1,2-L-Arap linkage in rhamnogalacturonan II; L-Rha-α-1,4-D-GalA linkage in rhamnogalacturonan |
| 86 | GH109 | 6 | 4 | α-N-acetylgalactosamine; A antigen of blood group |
| 87 | GH110 | 2 | 1 | Terminal α(1,3)-linked galactose residue on blood group B/O |
| 88 | GH115 | 3 | 5 | 4-O-methyl D-glucuronic acid sidechains of native xylan polysaccharides; 4-O-methyl-D-glucuronic acid and non-methylated D-glucuronic acid from xylan and xylo-oligosaccharides |
| 89 | GH116 | - | 1 | glucosylceramides, N-acetyl-glucosaminides, and xylosides |
| 90 | GH117 | 8 | 1 | α-1,3-L-(3,6-anhydro)-galactoside |
| 91 | GH123 | 4 | 3 | non-reducing terminal β-GalNAc linkage in glycosphingolipids |
| 92 | GH125 | 8 | 8 | α-1,6-linked non-reducing terminal mannose residues |
| 93 | GH 127 | 14 | 16 | β-L-arabinofuranoside in saccharides and amino acid glycoconjugates and hydroxyproline-rich glycoproteins |
| 94 | GH 130 | 1 | 3 | β-mannosides |
| 95 | GH 136 | 1 | - | lacto-N-bioside; Lewis antigen a/b specificity |
| 96 | GH 137 | 1 | 1 | β-L-arabinofuranoside in rhamnogalacturonan II |
| 97 | GH 138 | 2 | 1 | (GalA-α1,2(GalA-β1,3)(2MeXyl-α1,3-Fuc-α1,4)Rha-α1,3-Api) of chain A from the pectic polysaccharide rhamnogalacturonan II |
| 98 | GH 139 | 1 | 1 | 2-O-methyl-L-fucose-α-1,2-D-Gal*p* linkage in chain B of the complex pectin rhamnogalacturonan-II |
| 99 | GH 140 | 3 | 1 | α(1,2)-apiose on the sidechains A and B of the complex glycan rhamnogalacturonan |
| 100 | GH 141 | 3 | 1 | fucosodic linkage in 2-O-methyl-D-xylosyl-α-1,3-L-fucosyl-α1,4-L-Rhap of the chain B of rhamnogalacturonan II |
| 101 | GH 143 | 1 | 1 | β-1,2-glucooligosaccharide |
| 102 | GH 144 | 4 | 5 | β-1,2-glucooligosaccharides |
| 103 | GH 145 | 2 | 2 | rhamnose linked α-1,4 to glucuronic acid (Rha-GlcA) in the complex arabinogalactan protein (AGP)  (Polysaccharide Lyase Family 42) |
