# Supplementary material for: Effects of Prebiotic Dietary Fibers on the Stimulation of the Mucin Secretion in Host Cells by In Vitro Gut Microbiome Consortia
Source: Foods. 2024 Oct 8;13(19):3194. doi: 10.3390/foods13193194 (PMC11475894; doi:10.3390/foods13193194)
Supplement: Supplementary file 1 [file foods-13-03194-s001.zip › Supplementary data 3.docx]

**Supplementary data 3**


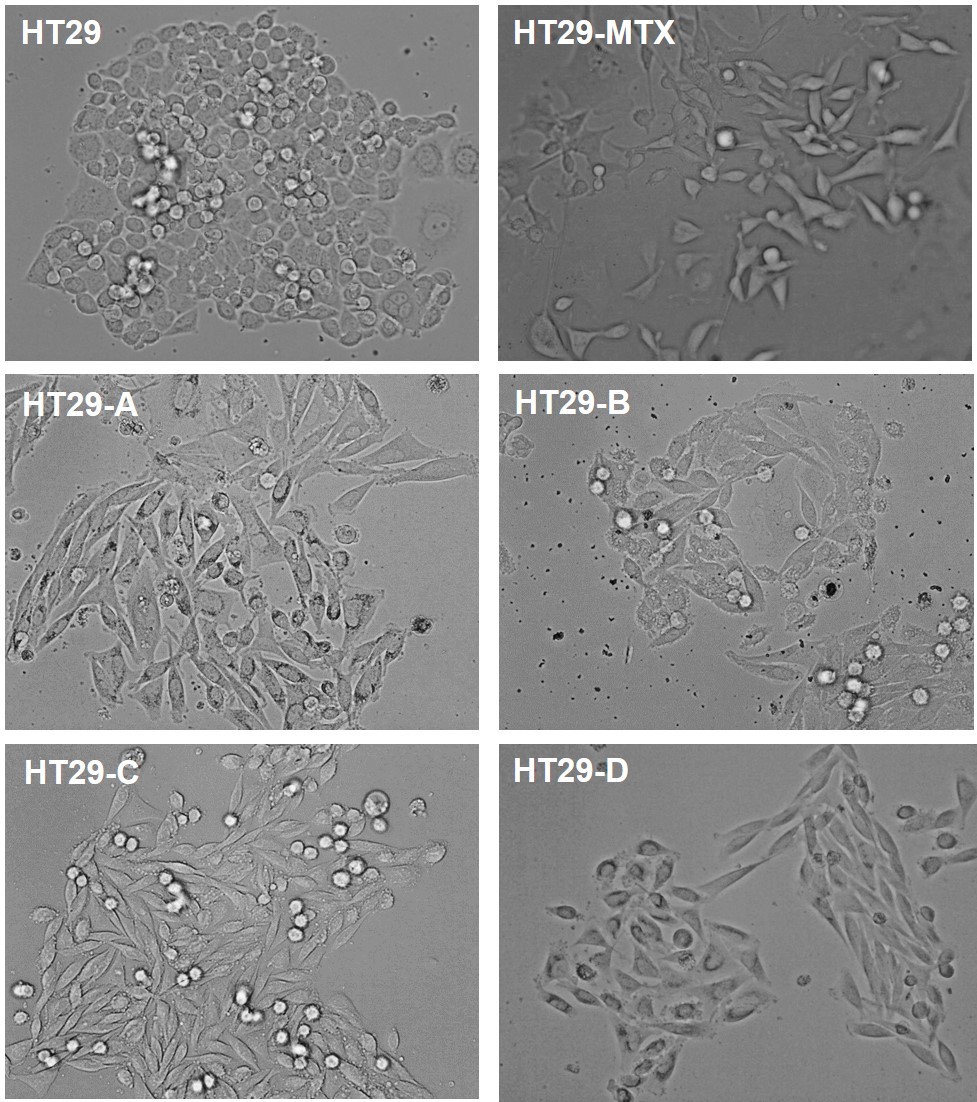


**Fig. S1.** Phase contrast microscopy analysis for the morphological changes for the adapted HT29 cell lines. HT29 is a native cell as a negative control; HT29-MTX is a methotrexate-stimulated cell (a positive control); HT29-A, -B, -C, and –D were the adapted cell lines exposed to the bacteria-free SCFAs supplemented with *H. erainceus* (A), *S. crispa* (B), oat (C), or barely (D), respectively.
